# Spatial analysis of HPV‐associated cervical intraepithelial neoplastic tissues demonstrate distinct immune signatures associated with cervical cancer progression

**DOI:** 10.1002/path.70002

**Published:** 2025-12-09

**Authors:** Gianna Pavilion, Hani Vu, Zherui Xiong, Thi Viet Trinh Dang, Blake O'Brien, Michael Walsh, Andrew Causer, Janin Chandra, Quan Nguyen, Ian H Frazer

**Affiliations:** ^1^ Institute for Molecular Bioscience The University of Queensland St Lucia QLD Australia; ^2^ QIMR Berghofer Herston QLD Australia; ^3^ Frazer Institute, Faculty of Health, Medicine and Behavioural Science The University of Queensland Woolloongabba QLD Australia; ^4^ Sullivan Nicolaides Pathology Bowen Hills QLD Australia; ^5^ Dermatology Research Centre The University of Queensland Brisbane QLD Australia; ^6^ QIMRB National Centre for Spatial Tissue and AI Research QIMR Berghofer Herston QLD Australia

## Abstract

Cervical cancer remains the fourth most common cancer affecting women worldwide, and incidences of other HPV‐related cancers continue to rise. For the development of effective prevention strategies in high‐risk patients, we aimed to better understand the roles of inflammatory pathways and the tumour microenvironment as the main driver of progression to malignancy in HPV‐infected tissues. We analysed the spatial organisation of seven samples of HPV+ high‐grade squamous intraepithelial lesion (HSIL) and cervical intraepithelial neoplasia 3 (CIN3), comparing tumour heterogeneity and immune microenvironments between premalignant (neoplastic) and adjacent cervical tissues. We observed evidence of immune suppression within the neoplastic regions across all samples and identified distinct immune clusters for each dysplastic lesion. Previous single‐cell data analyses in an HPV16 E7 oncoprotein‐driven transgenic mouse model suggested a potential role for IL34‐CSF1R signalling in immune modulation, where low IL34 expression was associated with Langerhans cell dysfunction, and, in cervical cancer, with poor patient outcome. Here we observed that IL34‐CSF1R coexpression was absent within HPV‐associated neoplastic regions, but present in adjacent normal tissue regions. Additionally, we identified enrichment of an M2 gene signature in neoplastic regions, while adjacent tissue was enriched with a proinflammatory M1 gene signature. Our findings provide biopathological insights into the spatial cellular and molecular mechanisms underlying HPV‐associated cervical cancer immune regulation and suggest a strategy to modulate the immune system in HPV‐positive neoplastic cervical and other tissues. © 2025 The Author(s). *The Journal of Pathology* published by John Wiley & Sons Ltd on behalf of The Pathological Society of Great Britain and Ireland.

## Introduction

Despite significant advances in biomedical research and public health interventions, cervical cancer persists as a major global health concern, ranking as the fourth most prevalent cancer among women worldwide [1, 2]. Moreover, the incidence of human papillomavirus (HPV)‐driven anogenital and oropharyngeal cancers is on the rise globally, affecting both men and women [3, 4, 5]. Traditional histopathological examinations of tissue sections, while informative, lack the resolution to provide a comprehensive understanding of the complex genetic and immune‐spatial landscape of cervical lesions. Current knowledge of cervical intraepithelial neoplasia Stage 3 (CIN3) morphology primarily relies on insights derived from the study of E6 and E7 oncoproteins expressed by oncogenic high‐risk HPV types [6, 7, 8]. There remains much ambiguity surrounding the mechanisms underlying HPV persistence, immune dysregulation, and the progression from low‐ to high‐risk intraepithelial lesions [9]. Thus, a deeper understanding of these mechanisms is crucial for the development of effective prevention and treatment strategies.

One potential mechanism of immune suppression involves the dysregulation of cytokine signalling pathways that control immune cell recruitment and function. In particular, the interactions between interleukin‐34 (IL34) and its receptor, colony stimulating factor‐1 receptor (CSF1R), have emerged as key modulators of immune responses in various cancers [10, 11]. CSF1R is expressed exclusively on mononuclear phagocyte cells [12]. IL34 has been identified as an alternative ligand for CSF1R, promoting monocyte survival [13]. While IL34 is expressed in various tissues, it is most abundantly found in the skin and brain, where it plays a crucial role in the maintenance and development of Langerhans cells (LCs) and microglia, respectively [14, 15]. IL34‐CSF1R signalling can lead to activation of various pathways, including STAT, AKT, and ERK1/2, caspase, and autophagy, which in turn trigger cell proliferation, differentiation, migration, and cytokine production [16].

The role of IL34 in cancer progression is not fully understood, and whether it has a protective or detrimental effect depends on the tissue and cancer types [17]. However, in the progression of cervical intraepithelial lesions to invasive cancer, our research identified a continuous decline in *IL34* gene expression, which also correlated with poor patient prognosis [18]. We previously demonstrated in a transgenic mouse model of HPV16 E7‐mediated epithelial hyperplasia that *IL34‐CSF1R* coexpression was absent between hyperplastic keratinocytes and LCs [18]. This absence was associated with an imbalance in LC cell states, characterised by a depletion of mature immune‐stimulatory LCs and an enrichment of semimature immune‐inhibitory LCs [18]. In line with this, we have found that LCs of HPV16 E7 mediated epithelial hyperplasia were dysregulated in phenotype, maturation, and function [18, 19, 20, 21, 22].

These findings suggest that HPV‐driven epithelial hyperplasia may impair LC function and contribute to immune evasion, potentially facilitating malignant progression [18]. Additionally, Strachan *et al* [23] demonstrated in a mouse model that inhibition of the CSF1R signalling pathway, which regulates recruitment of tumour‐associated macrophages (TAMs), reduces TAM turnover and tumour growth, while increasing T‐cell infiltration. These data support continuous CSF1R blockade as a potential anticancer therapeutic strategy. These murine models have also been valuable in simulating the pathophysiology of high‐grade squamous intraepithelial lesions (HSIL) and furthering our understanding of the role of LCs and TAMs in carcinogenesis [23].

Examining the spatial distribution of immune cells and neoplastic epithelial cells within human CIN3 lesions can yield insights unattainable with traditional methods or murine models. The advent of spatial and multi‐omics approaches offers unprecedented resolution and contextual depth for studying the tumour immune microenvironment [24]. Recent single‐cell analyses have uncovered key molecular signatures, specific signalling pathways, and distinct cellular communities within cervical cancer [25, 26, 27, 28, 29]. However, a comprehensive spatial analysis of human CIN3/HSIL samples is lacking.

In this study we aimed to better understand the role of inflammatory pathways and the tumour microenvironment as key drivers of progression to malignancy in HPV‐infected tissues. To achieve this, we leveraged spatial methodologies to conduct a detailed immuno‐spatial analysis of human CIN3 samples. Our investigation explored ligand‐receptor pair interactions, specifically focusing on the coexpression of *IL34* and *CSF1R* in HPV+ neoplastic human tissues. By drawing mechanistic parallels to other HPV‐driven cancers, our findings reveal novel insights into the immune landscape of CIN3 and identify a potential alternative pathway for therapeutic intervention.

## Materials and methods

### Ethics approval and sample selection

This study was approved by The University of Queensland Human Research Ethics Committee A (Project No. 2022/HE001601) and conducted in accordance with the National Statement on Ethical Conduct in Human Research (2007, current version). Samples were selected by pathologists at Sullivan Nicolaides Pathology (SNP) and corresponding p16 immunochemistry staining was performed on each biopsy to confirm the diagnosis of CIN3 (supplementary material, Figure S1). Ultimately, eight samples were selected based on the criteria of the absence of initial patient treatment and the presence of prominent p16 staining affecting more than two‐thirds of the epithelium. Additionally, all lesions originated within the transformation zone of the cervix. Notably, the natural history of CIN3 is influenced by several known factors, including HPV genotype, immune competence of the host, duration of the infection, as well as by suspected or less well‐known factors, such as the local microbiome and/or components from male ejaculate. Therefore, some variability in any investigation designed to map associations of HPV‐induced CIN3 would be expected. Nevertheless, on comparing the spatial mapping on tissue regions, we observed that CIN3 regions are more consistent with each other across patients compared to other epithelial regions, such as non‐SCC and SCC areas. We designed the cohort by selecting lesions that all arose in the transformation zone of the cervix. We also selected large biopsies from LLETZ (Large Loop Excision of the Transformation Zone) cohort, in which CIN3 regions could be selected for spatial profiling. To enhance generalisability, we also compared our data with independent datasets generated by other groups, although spatial transcriptomics data resources are very limited. The results of comparisons with external datasets are presented in the supplementary material, providing supporting evidence for the conclusions drawn from our patient cohort. Histopathological assessment was further conducted by a qualified pathologist on a matched reference H&E tissue section to define the specific region of interest within a 6.5 mm × 6.5 mm Visium capture area.

### Tissue preparation: H&E staining and imaging

Eight 5‐μm formalin‐fixed, paraffin‐embedded (FFPE) CIN3 samples, cut on SuperFrost slides, were provided by SNP. In accordance with the 10X Genomics Demonstrated Protocol (#CG000520), tissue sections were deparaffinised and stained in Mayer's haematoxylin (Dako, Glostrup, Denmark) for 5 min and eosin (Sigma, St. Louis, MO, USA) for 1 min [30]. Imaging was performed using a Leica Aperio XT Brightfield Automated Slide Scanner (Leica Microsystems, Wetzlar, Hesse, Germany) with 40× magnification. Following imaging, sections were destained and decrosslinked in preparation for library construction.

Using these H&E images, a pathologist was asked to outline regions of nondysplastic squamous epithelium (non‐CIN3), early invasive squamous cell carcinoma (SCC), and CIN3 neoplastic regions across the tissue section (supplementary material, Figure S1). The annotations were performed blinded to the p16 immunochemistry staining results.

### Immunofluorescent staining

Immunofluorescent staining was performed sequentially for CD206, CD86, and p16 using the Opal‐TSA system (AKOYA Biosciences, Menlo Park, CA, USA) [31]. In brief, adjacent CIN3 FFPE sections were deparaffinised with xylene and rehydrated through a gradient ethanol wash. Heat‐induced epitope retrieval was performed using Target Retrieval Solution, Citrate pH 6 (Dako, Glostrup, Denmark; S236984‐2) at 100 °C for CD206 and CD86; and with EDTA pH 9.0 at 105 °C for p16. Background Sniper (Biocare Medical, Pacheco, CA, USA; BS966) was used as blocking buffer to minimise unspecific binding. Primary antibodies were sourced as follows: CD206 (Abcam, Cambridge, UK; ab64693); CD86 (Abcam; ab243887), and p16 (BD Biosciences, San Jose, CA, USA; G1775‐405). The Tyramide Signal Amplification (TSA) was applied using Opal 520 (AKOYA Biosciences; AKO‐FP1487001KT) for CD206, Opal 690 (AKOYA Biosciences; AKO‐FP1497001KT) for CD86, and Opal 570 (AKOYA Biosciences; AKO‐FP1488001KT) for p16.

### Library generation and sequencing

Sequencing libraries were constructed using the Visium CytAssist Spatial Gene Expression for FFPE, Human Transcriptome, 6.5 mm kit (10X Genomics, Pleasanton, CA, USA; PN #1000520) according to the Visium Spatial Gene Expression User Guide Rev C (10X Genomics; #CG000495). A total of 10–13 PCR cycles were employed for the amplification of the final Visium libraries. A qualitative assessment of each library was performed. Paired‐end dual‐indexing was performed on the NovaSeq 6,000 platform [32] using the following protocol: Read 1: 28 bp, i7: 10 bp, i5: 10 bp, and Read 2: 50 bp. Illumina (San Diego, CA, USA) sequencing base call data (BCL) was demultiplexed using the Bcl2Fastq conversion software (v2.20) [32]. The SpaceRanger computational pipeline (10X Genomics; V2.0) was used to align the sequencing data to the GRCh38 human reference genome and map the data to the associated spatial coordinates of the H&E image, determined by spatial barcode information [33].

### Quality control, normalisation, and batch correction

#### Quality control

Various processing issues during sample preparation such as tissue dissociation, folding, and other technical issues may lead to artefacts. These may translate into the data as variations in sequencing depth and batch effects. Mapped and aligned data were imported into the R package Seurat (v5.1.0) for data analysis [34]. Genetic expression levels were determined based on the number of unique molecular identifiers [32]. Outliers and low‐quality reads were removed by filtering spots at a fixed threshold of <100 counts per spot. Sparse genes expressed in <3 spots were also removed.

Quality assessment of sample VLP90_D revealed a substantial proportion of spots with a high percentage of mitochondrial transcripts (supplementary material, Figure S2A), indicative of potential sample degradation or compromised cell integrity. Furthermore, spatial visualisation and quantification revealed minimal expression of *CDKN2A* across the spots within this sample (supplementary material, Figure S2A,B). No correlation between p16 abundance and *CDKN2A* expression was found, suggesting possible exhaustion of the CIN3 region within the Visium sample. Due to these quality control metrics and the sample selection criterion not being met, sample VLP90_D was excluded from downstream analyses.

#### Data normalisation

The Seurat *SCTransform* function was applied to normalise and scale filtered data. The function employs a regularised negative binomial regression, allowing biological variation to be retained within genetic expression profiles while eliminating technical variation and discrepancies among sequencing runs [34]. Preliminary selection of the top 3,000 variable genes attained through this function was utilised for downstream analysis.

#### Batch correction and data integration

The normalised data were corrected for batch effect using Seurat's *IntegrateData* function. The top 3,000 variably expressed genes selected for each sample were leveraged to compute anchors through Canonical Correlation Analysis [35] and Mutual Nearest Neighbors (MNN) analysis, allowing technical differences to be mitigated [36], was conducted using the first 30 principal components (PCs) [35, 36]. The number of PCs was selected based on elbow plot visualisation. Uniform Manifold Approximation and Projection (UMAP) embedding were generated to visualise the results.

### Clustering and spot type annotation

Spatial transcriptomics (ST) techniques/technologies such as 10x Visium allow for deeper analysis of gene expression patterns, whilst also preserving tissue morphology. Unsupervised clustering of the integrated dataset was performed using Seurat's *FindClusters* function, in which spots with similar gene expression profiles are grouped together using a shared nearest neighbour (SNN) modularity optimisation‐based clustering algorithm (*FindNeighbors*). Clusters were determined using Louvain clustering, at a resolution of 0.5. Seurat's *FindAllMarkers* function was used to perform differential gene expression (DGE) analysis and compare each cluster against all other clusters by leveraging the Wilcoxon rank sum test [34]. Marker genes were identified using a stringent filtering criterion, including minimum percentage expression of 0.25, a log fold change threshold of 0.25, and an adjusted *p* value of 0.05. The top 50 marker genes significantly enriched were used to infer potential cell types and annotate individual clusters using EnrichR and the PanglaoDB gene database [37, 38, 39, 40].

### Pseudo‐bulk processing and differential gene expression

To perform a more comprehensive DGE analysis between clusters, gene expression levels from individual spots within each cluster were aggregated. Using the *scater* package, spots were pseudo‐bulked by summing the raw UMI counts for each gene within each cluster [35]. To increase the statistical power, the clusters were pooled into ten pseudo‐replicates. Using *filterByExpr* and *calcNormFactors*, implemented within the *edgeR* package, genes with insufficiently low counts in the pseudo‐bulked clusters were removed and read counts were normalised [41]. Subsequently, DGE analysis was conducted using the *edgeR* framework, incorporating elements of linear modelling from the *limma* package for robust statistical inference, allowing genes with significantly altered expression levels between each cluster to be identified [42]. *edgeR* further fits a negative binomial model using *glmQLFit* to account for overdispersion and employs *glmTreat* to identify differentially expressed genes (DEGs) based on a false discovery rate (FDR)‐adjusted *p* value <0.05 and a logFC threshold of 1.2. All upregulated marker genes were included in the analysis, except for those in cluster 5, where only the top 50 genes ranked by FDR were considered. To interpret the results, a heatmap was generated using *pheatmap*, providing a visual representation of the expression patterns of DEGs across each cluster.

### Subclustering analysis

Additional subclustering analysis was performed on all spots identified as cluster 8. Using a similar method described above, Louvain clustering was implemented for each sample individually, based on the first 30 PCs at a resolution of 0.3. Subclusters were then grouped into either CIN3, non‐CIN3, SCC, or Other (p16+) classes based on the expression of key marker genes, CIN3; *SERPINB13*, *SERPINB5*, *CLDN1*, *TP63*, *CDKN2A*, *TNS4*, and *DSC3*, non‐CIN3; *KRT6C*, *GJB6*, *SBSN*, *KRTDAP*, and *KRT6B*, and SCC; *CASP14*, *PSORS1C2*, *DNAH17*, *SERPINB12*, *SLC44A5*, *TCHH*, *PNLDC1*, *DIAPH3*, *CPA4*, and *CALML5* (supplementary material, Table S1). These genes were selected based on a consensus set of DEGs specific to each cluster, calculated using the methods described above.

### Cell cycle scoring

Cell cycle phase assessment was performed using Seurat's *CellCycleScoring* function. Predefined gene sets specific to human were employed to identify cells in S and G2/M phases as per the scoring strategy previously described by Tirosh *et al* [43].

### Immune cell type identification

Based on the previous literature, manually defined immune marker gene sets were used to determine significantly different expression patterns and perform gene set enrichment analysis (supplementary material, Table S2). Using *AddModuleScore*, coexpression patterns of gene set markers were identified and adjusted by subtracting the aggregated expression across the whole tissue sample [43]. Immune cell populations were identified using the following markers: T‐reg (*CD4*, *TNFRSF18*, *IL2RA*, *FOXP3*, *TGFB1*, and *IL10*) [44, 45, 46], antiinflammatory T cells (*IL10*, *IL12A*, *IL12B*, *IL22*, *IL37*, *IL1F10*, *TGFB1*, *IL4*, *IL11*, and *IL13*) [46, 47], proinflammatory T cells (*IL10*, *CSF3*, *IL1A*, *IL1B*, *IL6*, *IFNG*, *IL4*, *IL5*, *IL13*, *IL36B*, *IL36G*, *IL36A*, *CXCL8*, *TNF*, and *IL18*) [46, 47], M1 macrophages (*CD80*, *IFNG*, *IL1B*, *IL6*, *TNF*, *CCL2*, *FCGR3A*, *FCGR2A*, *FCGR1A*, *IL12B*, *IL23A*, *MARCO*, and *CD86*) [46, 47, 48] M2 macrophages (*VTCN1*, *CD36*, *CD200R1*, *CD163*, *MRC1*, *CD209*, *CLEC10A*, *CLEC7A*, *CXCR1*, *CXCR2*, *MSR1*, *ARG1*, *NOS2*, *RETNLB*, *HMOX1*, *PPARG*, *CD274*, *IL1RN*, *TGM2*, *ACTG1*, *TIMP1*, *SPHK1*, *CXCL1*, *MERTK*, *ITGAV*, *IL1A*, *VEGFA*, *IVNS1A BP*, *CXCL8*, *DYSF*, *EHHADH*, *RIF1*, and *LIG4*) [46, 47, 48], and Langerhans cells (*CD207*, *CD1A*) [38, 49, 50].

### Macrophage activity score

Macrophage activity within clusters 8, 9, and 10 was calculated using the predefined marker gene list for M1 macrophages and M2 macrophages, as listed above. The difference in macrophage activity between the two cell states was calculated using the following equation:

$$M2-M1=\left[\left(\sum \left(\mathrm{GE}\_m_{2}\right)/n\right)-\left(\sum \left(\mathrm{GE}\_c\right)/k\right)\right]-\left[\left(\sum \left(\mathrm{GE}\_m_{1}\right)/n\right)-\left(\sum \left(\mathrm{GE}\_c\right)/k\right)\right]$$

$\mathrm{GE}\_m_{1}$: expression value of genes in M1 macrophage module group. $\mathrm{GE}\_m_{2}$: expression value of genes in M2 macrophage module group. GE_c: expression value of genes in the control group for module. n: number of genes in each module group. k: number of genes in the control group.

### IL34‐CSF1R *versus* tumour expression

The relationship between IL34‐CSF1R coexpression and the tumour microenvironment was investigated by comparing the spatial expression patterns of *IL34*‐*CSF1R* and cervical cancer gene markers (*SERPINB3*, *TP63*, *KRT5*, and *CDKN2A*) [25]. To determine meaningful expression, only data points with a module score above a threshold of 0.1 were considered, otherwise the spots were labelled as ‘None’. Subsequently, using a module‐based data partitioning method, the remaining spots were categorised into ‘IL34‐CSF1R’ or ‘Tumour’ based on their dominant module score.

### Cell–cell interaction analysis

Using stLearn, a package designed for downstream ST analysis, cellular communication is inferred through ligand‐receptor interactions within and between spots [51]. From a list of known ligand‐receptor pairs, the package calculates the genetic expression for each ligand and receptor and determines the statistical difference of the ligand‐receptor coexpression. In comparison to the background distribution of all random gene–gene pairs, the *p*‐value is determined by the cumulative occurrence where the ligand‐receptor pair has scored higher than other random combination pairs. The spatial proximity of each ligand‐receptor pair is also taken into consideration, with intermediate neighbouring cells more likely to interact. By combining genetic expression and spatial information, stLearn is able to rank and assess the significance of ligand‐receptor pair interactions and interpret potential cell–cell communication.

### Trajectory and pseudotime analysis

The R package Monocle3 was utilised to perform trajectory analysis. This package uses low‐dimensionality UMAP space to group transcriptionally similar spots or cells together and form a path or trajectory between these cell groups. This trajectory is then resolved to detect branches and convergence regions throughout the path [52]. Using Monocle3, all spots from our seven samples were processing with 30 PCs and aligned to remove batch effects between samples. Spots were then projected into UMAP space and grouped with Louvian clustering. The trajectory path was generated using learn_graph. Based on this trajectory, the node ‘Y_146’ was selected as the root to perform pseudotemporal ordering (pseudotime), as it was localised within the CIN3 cluster. In brief, pseudotime analysis involves the ordering of spots or cells along a path based on changes in their transcriptional profile [52]. Pseudotime statistics were calculated using order_cells in Monocle3. The function graph_test was then used to identify genes that follow this pseudotime trajectory based on Moran's I test statistics [52].

### Oropharyngeal squamous cell carcinoma (OPSCC) analysis

*IL34*‐*CSF1R* expression was further analysed using a public oropharyngeal squamous cell carcinoma (OPSCC) dataset to explore potential similarities in HPV+ carcinogenesis [24]. A module score for the expression of OPSCC HPV+ tumour marker genes (*SOX4*, *SLC2A1*, *KRT8*, *EIF4A2*, *SNAI2*, *CA12*, *HSPH1*, *ANGPTL4*, *FAM162A*, *PFKP*, *TGFB1*, *UHRF2*, *PSIP1*, *MYC*, *RCL1*, *KDM4C*, *ACTL6A*, *CHAF1A*, *DEK*, *ECT2*, *POLR1D*, *GAS1*, *PLOD2*, *DDIT4*, *IL33*, *ABCC5*, *ALCAM*, *CALCRL*, *MKNK2*, and *MT2A*) and *IL34*‐*CSF1R* coexpression was determined and plotted using *SpatialDimPlot*. Gene set expression was compared with pathological annotations associated with OPSCC HPV+ samples [24].

### External HPV+ cervical spatial transcriptomics dataset

The public dataset GSE208654 (https://www.ncbi.nlm.nih.gov/geo/query/acc.cgi?acc=GSE208654) containing spatial transcriptomics data of HPV+ cervical tissue, one normal (N), one precancerous (HSIL), and two cervical cancer (SCC and ADC) was also used to explore the *IL34*‐*CSF1R* signature across disease stage [53]. Enrichment of CIN3, oncogenes, and SCC gene sets (supplementary material, Table S1) were also compared across all four samples.

## Results

### Unsupervised identification of 11 gene expression clusters across seven CIN3+ patients

**Figure 1:**
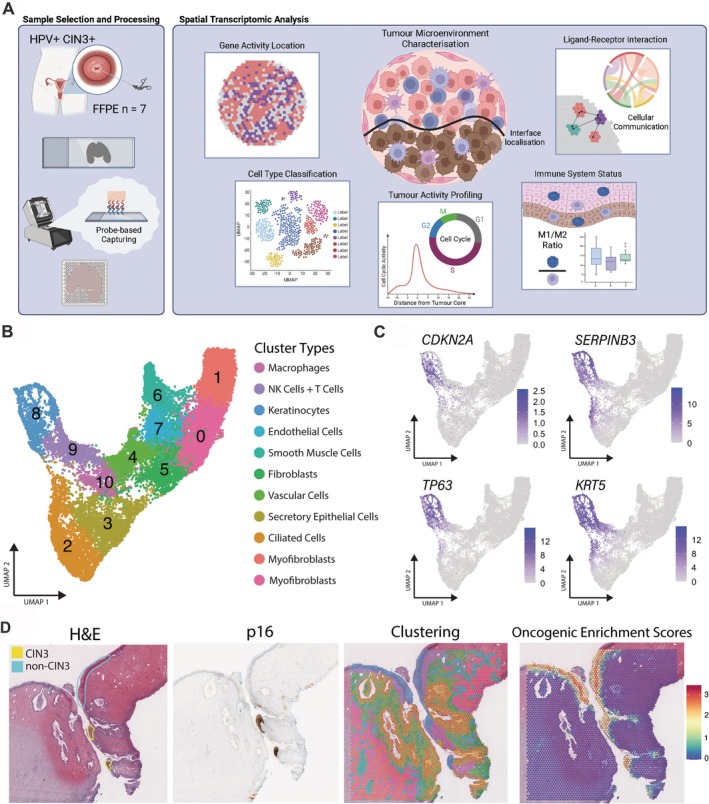
Characterisation of the immunospatial landscape of seven HPV+ CIN3+ samples. (A) Schematic of the experimental design and spatial transcriptomic analysis. (Figure created with BioRender.com). (B) Integrated UMAP displaying cell type distribution across the seven samples. (C) Feature plots of known cervical cancer oncogenes: *CDKN2A*, *SERPINB3*, *TP63*, and *KRT5* (Ou et al., 2021 [25]). (D) H&E image with pathological annotations outlining CIN3 and nondysplastic squamous epithelium regions (non‐CIN3) in yellow and blue, respectively, p16 immunohistochemistry staining for HPV+ CIN3+ lesion validation, spatial clustering with corresponding cluster colours as in panel (B), and identification of CIN3+ region using cervical cancer oncogene coexpression for a representative sample (VLP89_A).

**Figure 2:**
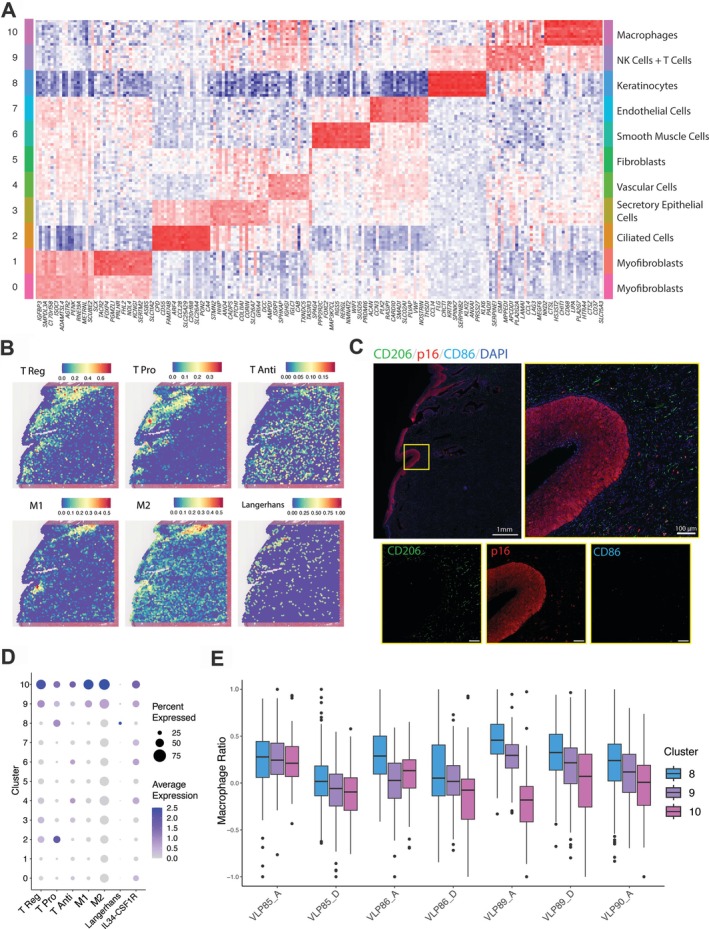
Analysis of immune cell types and identification of a distinct immune cluster. (A) Expression heatmap of the top 10 upregulated marker genes for each cluster. (B) Spatial mapping of immune cell module scores for a representative sample (VLP86_A). (C) Immunofluorescence staining on adjacent tissue sections for a representative sample (VLP86_A) to evaluate the CIN3+ lesion (p16) and the presence of M1 (CD86) and M2 (CD206) macrophages. Nuclei were stained using DAPI. Fluorescent signals for the magnified region, highlighted by the yellow box, shown in greyscale for each channel. Scale bars, 100 μm. (D) Dot plot of gene expression scores based on canonical immune cell markers. (E) M2 to M1 module score ratio in clusters 8, 9, and 10 for all seven samples.

Utilising the comprehensive transcriptome‐wide profiling capabilities of Visium CytAssist (10X Genomics), the spatially resolved gene expression profiles of CIN3+ lesions were captured using a probe‐based approach (Figure 1A). Across the seven samples, the transcriptome for 21,054 barcoded 55‐μm diameter spots were measured, with each spot containing ~1–10 cells. A median of 4,422–5,730 genes were detected per spot, demonstrating transcriptionally active cells across the tissue. Unsupervised clustering revealed 11 distinct clusters across seven samples (Figure 1B). In addition to transcriptional differences (Figure 1C), these clusters displayed distinct spatial distributions across the tissue, highlighting key spatial patterns that appeared consistently across pathological annotations, immunohistochemistry staining, and spatial transcriptomic data (Figure 1D; supplementary material, Figure S1). Using significantly expressed genes, each cluster was then annotated based on gene set enrichment analysis using the PanglaoDB cell type database (Figures 1B and 2A) [38]. The identified clusters were annotated with major cell types as myofibroblasts (0 and 1), ciliated cells (2), secretory epithelial cells (3), vascular cells (4), fibroblasts (5), smooth muscle cells (6), endothelial cells (7), epithelial cells and keratinocytes (8), natural killer cells and T cells (9), and macrophages (10) (Figure 2A). Although interpatient heterogeneity was evident in the distribution of cell types between samples, all samples exhibited similar patterns in cluster formation and gene expression (supplementary material, Figure S1). The spatial distribution of these clusters and key marker genes further validated the accuracy of our cell type identification (supplementary material, Figures S1 and S3).

### Mapping neoplastic regions highly enriched for oncogenes

We observed that cluster 8, representing keratinocytes, contained the highest number of significant DEGs when compared to the other clusters, indicating a unique microenvironment with distinct cell activity (Figure 2A). Genes most upregulated by cluster 8 included *KRT78*, *SERPINB2*, *ANXA1*, and *CLIC3* (Figure 2A). Additional DEGs that were overexpressed in cluster 8 included several known canonical cervical cancer oncogenes [25] – *CDKN2A*, *SERPINB3*, *TP63*, and *KRT5* (Figure 1C). The spatial expression patterns of this combined oncogene set showed particularly high colocalisation with cluster 8, suggesting the presence of neoplastic epithelial cells in this region (Figure 1D; supplementary material, Figure S1). Encoding tumour suppressor proteins p16INK4a and p14arf, the spatial distribution of *CDKN2A* (p16) was of particular interest. Within the region of cluster 8, this gene displayed greater heterogeneity within each sample, when compared to the individual expression patterns of *SERPINB3*, *TP63*, and *KRT5* (supplementary material, Figure S3). This observation was further supported by pathologist annotations and p16 staining, indicating key regions of neoplastic activity (supplementary material, Figure S1).

### Subclustering of epithelial regions reveals specific neoplastic hotspots

To further profile the heterogeneity observed within cluster 8, subclusters were generated from this region for each individual sample (supplementary material, Figure S4A). DGE analysis revealed a consensus set of genes corresponding to four tissue subtypes: ‘CIN3’, ‘non‐CIN3’, ‘SCC’, and ‘Other’ (supplementary material, Figure S4B,C). For example, subclusters identified as non‐CIN displayed significantly reduced expression of *CDKN2A* and elevated levels of *KRT6B* and *KRT6C* (supplementary material, Figure S4C). Comparatively, CIN3 subclusters displayed a significant upregulation of *CDKN2A*, *TP63*, *SERPINB13*, and *CLDN1* (supplementary material, Figure S4C). Spatially, these subtypes, highly correlated with pathologist annotations and p16 staining, in particular the CIN3 regions (supplementary material, Figures S1 and S4B), suggesting strong oncogenic activity was present within cluster 8. Cell cycle scoring demonstrated high proportions of spots in the S phase within this defined neoplastic region, indicating active cell replication, compared to surrounding spots found mainly in G1 and G2M phases (supplementary material, Figure S4E). Each sample displayed varying compositions of epithelial subtypes, emphasising the high complexity of these neoplastic microenvironments.

### Unique neoplastic communities displayed heightened immune cell activity

Gene expression analysis revealed prominent immune cell activity within cluster 10, based on canonical marker genes (supplementary material, Table S1). Based on DEGs, macrophages were observed as the dominant cell type (Figure 2A). In addition, T‐cell‐associated genes (supplementary material, Table S2) were enriched within cluster 10 in comparison to other clusters (Figure 2B,D). Spatial profiling demonstrated that the immune region (cluster 10) regularly neighboured the neoplastic region (cluster 8) across the seven samples (supplementary material, Figure S1). Analysis of expression signatures for key immune cell types (supplementary material, Table S2) revealed varying spatial distributions within cluster 10 (Figure 2B; supplementary material, Figure S5). Further subclustering of cluster 10 differentiated spots enriched with M1 and M2 macrophage gene signatures (supplementary material, Figure S6). Cell type proportions of these macrophages and other immune cell types displayed heterogeneity between samples (supplementary material, Figure S6). Using relative gene set enrichment scores (supplementary material, Table S2), M1/M2 macrophage ratios were calculated for each spot across individual tissue sections. The M2/M1 ratio was consistently higher in the neoplastic region (cluster 8) across all samples, indicating an immune‐suppressive macrophage microenvironment located specifically to the neoplasia (Figure 2E). This differential macrophage signature was corroborated by immunofluorescence staining of CD206 (M2) and CD86 (M1) in adjacent tissue sections, where M2 macrophages were more abundant around the CIN3 lesions (positive for p16) compared to M1 macrophages (Figure 2C). Conversely, the adjacent immune regions within cluster 10 had a comparatively low M2/M1 ratio across all samples, suggesting a more proinflammatory M1 macrophage microenvironment localised outside of the neoplastic region (Figure 2E). Cluster 9 also demonstrated high immune cell activity, with an intermediate M2/M1 ratio between that of the neoplastic region (cluster 8) and the immune region (cluster 10) (Figure 2E). Immune activity scores across all samples are summarised in the supplementary material, Figure S5. Cluster 9 also consistently bordered the neoplastic region and, at times, formed a complete barrier between cluster 8 and 10 when inspected spatially, highlighting a possible transitional zone between neoplastic and surrounding nonneoplastic regions (Figure 1D).

### Trajectory analysis identifies the transcriptional gradient of CIN3 neoplasia

To investigate the transitional zones of neoplasia identified above, we performed trajectory analysis on all spots (supplementary material, Figure S7). Following the alignment of spots based on their transcriptional profiles, a trajectory was resolved. A clear path was formed from defined CIN3 spots (cluster 8), followed by the bordering region (cluster 9), through to immune cells (cluster 10) (supplementary material, Figure S7A,B). This trajectory continued to other spot types, forming a various branch that splits similar lineages of cell types together, such as secretory epithelial cells (clusters 2 and 3), endothelial cells (cluster 7), smooth muscle (cluster 6), and fibroblasts (cluster 5) (supplementary material, Figure S7A). Using pseudotemporal analysis to score this trajectory, a clear gradient was identified (supplementary material, Figure S7B). Interestingly, this trajectory was generated based on transcriptomic variation alone; however, a clear spatial gradient was also observed across all samples (supplementary material, Figure S7C). This pseudotime trajectory displayed a gradient of neoplastic intensity radiating out from the CIN3 core, a degree of detail beyond the resolution of pathologist annotations (supplementary material, Figure S7C). This gradient could also distinguish between CIN3 regions and other epithelial areas (such as non‐CIN3 and SCC), further highlighting the complex nature of these CIN3 lesions (supplementary material, Figure S7C). This was validated based on the correlation of gene expression along the trajectory (supplementary material, Figure S7D). *CDKN2A* displayed a negative correlation against pseudotime compared to *IGF BP5*, which displayed increased expression at later pseudotimepoints (supplementary material, Figure S7D). Stromal cells such as fibroblasts, located at the end of the trajectory path, are known to secrete *IGF BP5* [54].

### Cell–cell interaction and ligand‐receptor pair analysis

**Figure 3:**
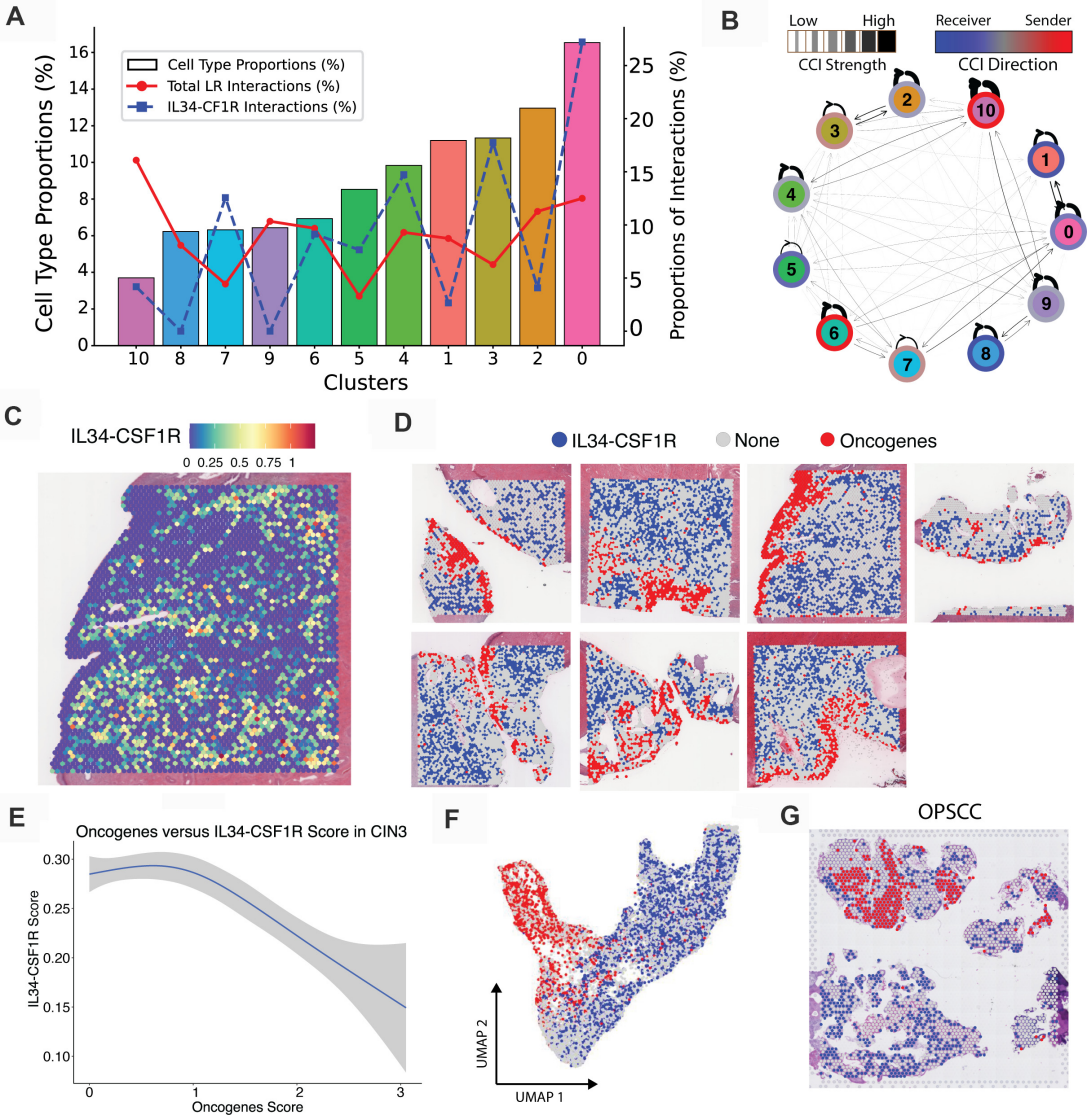
Spatial analysis of cell–cell interactions based on neighbourhood expression of ligand‐receptor pairs in CIN3+ samples and comparisons with external OPSCC samples. (A) Cell type proportions (bar plots) for each cluster across all samples with the percentage of ligand‐receptor interactions (line plots). Total ligand‐receptor interactions are indicated by the red line (proportion of spots with significant interactions among all spots) and the *IL34*‐*CSF1R* interactions as indicated by the blue line (significant *IL34*‐*CSF1R* interactions among significant interactions of all ligand‐receptor pairs). (B) Cell–cell interaction network displaying intra‐ and intercluster ligand‐receptor interactions with thicker lines representing stronger interactions and arrows indicate receiver/sender status of each cluster pair. (C) Spatial visualisation of *IL34*‐*CSF1R* coexpression module score (see Materials and methods section). (D) Cancer *versus IL34*‐*CSF1R* dominated region categorisation was determined by predominant module score expression of either cancer oncogenes, *IL34*‐*CSF1R* coexpression or neither within each spot for all seven samples. (E) Changes in *IL34*‐*CSF1R* interaction scores relative to cervical cancer module scores. (F) Integrated UMAP displaying distribution of spots categorised by higher coexpression tumour oncogenes (red) or *IL34*‐*CSF1R* across all samples (blue). (G) Comparison of cancer oncogenes *versus Il34*‐*CSF1R* spatial expression patterns in a different HPV+ cancer system, namely OPSCC tissues with the same category colour scheme as panel (D). The OPSCC data was generated by Causer *et al* [24].

We performed transcriptome‐wide cell–cell interaction analysis to reveal interaction hot spots and consistent interactions between ligand‐receptor pairs and cell type pairs across all samples [51, 55]. Cluster 10, enriched for immune cells, displayed the lowest cell‐type frequency across all samples (Figure 3A), but demonstrated the highest level of ligand‐receptor activity, predominantly intracluster interactions (Figure 3B). Interestingly, cluster 8, containing the neoplastic region, demonstrated lower CCI activity (Figure 3A), with most interactions occurring between either cluster 9 or with itself (Figure 3B). Notably, both cluster 8 and cluster 10 demonstrated high intercluster relationships with cluster 9, but almost no interactions occurred directly between clusters 8 and 10 (Figure 3B). Specifically, 61 ligand‐receptor interactions were observed between clusters 8 and 9, 75 interactions between clusters 9 and 10, and only four interactions between clusters 8 and 10 (supplementary material, Figure S8). The top molecule signalling between the neoplastic region (cluster 8) and the adjacent cluster 9 was midkine (*MDK*), a well‐recognised gene overexpressed by various human malignancies, including cervical cancer, and playing a role in cell growth, survival metastasis, migration, and angiogenesis [56]. The *S100A9*‐*TLR4* signalling axis has been implicated in inducing immunosuppressive myeloid‐derived suppressor cells [57], and TRAIL signalling, while being a death signal, has been implicated in cell migration and invasion, thereby promoting cancer progression [58]. The identified communication between the neoplastic region (cluster 8) and the immune region (cluster 10) suggests that the viral presence continuously induces a proinflammatory response through complement activity, type 1 interferon signalling, and *XCL2*‐mediated immune cell recruitment, while *APOE* signalling promotes immune suppression (supplementary material, Figure S8). Overall, these data highlight the complex molecular and spatial intricacies of immune recognition of virally‐induced abnormalities in the neoplastic region, as well as the signalling from this neoplastic region that suppresses immune activity and promotes its progression towards an invasive phenotype.

### Spatial gene expression patterns of *IL34* and *CSF1R* suggest their interactions and roles in tumour progression

Our previous research has identified *IL34* as a dysregulated and prognosis‐associated gene in CIN and cervical cancer [18]. We therefore aimed to define the spatial characteristics of *IL34* signalling in this dataset. The *IL34*‐*CSF1R* ligand‐receptor relationship was examined within clusters by comparing coexpression levels relative to cancer oncogene coexpression. The highest *IL34*‐*CSF1R* interaction activity was observed in cluster 0, followed by cluster 3 (Figure 3A). Notably, we observed the lowest coexpression of this ligand‐receptor pair within the neoplastic and adjacent transitional regions of cluster 8 and cluster 9 (Figure 3A). When plotted spatially, the coexpression of *Il34*‐*CSF1R* was seemingly absent in neoplastic regions (Figure 3C), where the expression of cancer oncogenes dominated (Figure 3D). A negative correlation was observed between coexpression of *IL34*‐*CSF1R* and cancer oncogenes (Figure 3E), with a separation of gene expression when visualised on the UMAP (Figure 3F). Importantly, though, our data suggest that the *IL34*‐receiving cells (LCs and macrophages) are not absent from these neoplastic regions that lack *IL34* (Figure 2B–D), indicating that other molecules may contribute to LC retainment, macrophage infiltration, and M2 polarisation. To determine whether the *IL34*‐*CSF1R* interaction pattern extended to another HPV‐mediated cancer type, we explored a publicly available oropharyngeal cancer sample [24], which demonstrated similarities in regard to the absence of *IL34*‐*CSF1R* coexpression in neoplastic regions (Figure 3G). These data are in line with our previous research demonstrating a loss of *IL34* in CIN, cervical cancer [18], in head and neck squamous cell carcinomas, and a murine model of HPV‐mediated epithelial hyperplasia [59, 60, 61], and spatially defines the restricted loss of *IL34* to the neoplastic regions, while maintaining signalling in adjacent tissue regions.

### Validation of gene expression patterns in external HPV+ cervical samples

The IL34‐CSF1R interaction in the context of CIN3 and cervical cancer progression was further investigated in an external HPV+ cervical spatial transcriptomics dataset, comprised of normal (N), precancerous (HSIL), and cervical cancer (SCC and ADC) samples (supplementary material, Figure S9). The gene signatures mentioned above (such as canonical cervical oncogenes, CIN3 and SCC gene sets) were used to isolate neoplastic squamous epithelia. Similar to our findings, the SCC gene set displayed the highest and most specific enrichment within the SCC sample, compared to low enrichment scores observed in other samples (supplementary material, Figure S9A,B). The CIN3 and oncogene gene set showed similar enrichment trends across other diagnostic groups with low enrichment scores in the normal sample and increasing significantly in HSIL and SCC samples (supplementary material, Figure S9A). Spatial distribution analysis confirmed these trends, illustrating regional enrichment of oncogene and SCC markers within HSIL and SCC tissue types (supplementary material, Figure S9B). Consistent with our current findings, an inverse relationship was observed between *IL34‐CSF1R* interaction activity and oncogene gene set enrichment across these samples, where increased oncogene enrichment correlated with decreased *IL34‐CSF1R* coexpression activity (supplementary material, Figure S9C).

## Discussion

Although cervical cancer incidences have decreased over the past decades, it remains a significant global health burden, with an estimated 604,127 new cases and 341,831 deaths worldwide in 2020 [2]. Despite the emergence of medical advancements, there remains ambiguity surrounding the mechanisms underlying HPV persistence and disease progression. We set out to conduct a detailed spatial analysis of human CIN3 samples, focusing on the complex interplay between immune cells and the tumour microenvironment, particularly the role of *IL34* and *CSF1R* in tumour progression, as proposed in previous research [18, 60, 61].

We defined the presence of a neoplastic region across our dataset, within cluster 8, characterised by DEGs, including known cervical cancer oncogenes. Cluster 8 was the most enriched for *KRT78* and *SERPINB2*, both of which have been shown to exhibit increased expression in HPV‐associated cervical cancers [62, 63]. Notably, elevated SERPINB2 expression has been linked to the severity of cervical lesions [63]. Furthermore, we observed an upregulation of *ANXA1*, which has been shown to modulate inflammatory properties by promoting macrophage differentiation to its protumoral M2 phenotype, promoting tumour progression [64, 65]. Additionally, *CLIC3* was significantly upregulated in this cluster, a gene associated with HPV‐induced neoplastic cervical lesions and contributing to poor prognosis in patients with cervical carcinoma [66, 67]. We also observed the enrichment of *CDKN2A* (p16), *SERPINB3*, *KRT5*, and *TP63*, which are well‐established biomarkers for HPV‐positive neoplasia and are typically expressed in tumour areas [25]. *KRT5* and *TP63* are known epithelial and basal markers, and their overexpression has been implicated in pathways and lineages that drive carcinoma progression [68, 69]. *CDKN2A* is a canonical proliferation marker that is also a well‐documented biomarker for HPV infection, with expression levels increasing from early‐ to late‐stage CIN, and furthermore to invasive cervical cancer [29, 70, 71]. Additionally, *SERPINB3* has recently been associated with poor prognosis in cervical cancer [72]. *SERPINB3* is believed to contribute to tumour progression by protecting cancer cells from immune surveillance, thereby facilitating therapeutic evasion [73]. The elevated expression of *SERPINB3* within the neoplastic region suggests a potential mechanism for immune escape and treatment resistance in this subset of cervical cancer cells. Subclustering and cell‐cycle analysis indicated that the neoplastic region was predominantly in the ‘S' or DNA replication phase, consistent with the proliferative nature of CIN3 lesions [74, 75].

We observed an immune cell cluster (cluster 10) consistently neighbouring the lesions in each sample. Cluster 10 exhibited heightened immune cell activity, particularly T cells, macrophages, and LCs, suggesting an active and localised immune activity at the neoplastic border. Further analysis showed that an M2 macrophage cell state was more prevalent in the neoplastic region compared to normal adjacent regions, aligning with previous investigations exploring the spatiotemporal expression of M1/M2 in cervical cancer tissues. This finding supports previous studies highlighting the immunosuppressive role of M2 macrophages in promoting tumour growth and progression [18]. We identified a clear trajectory that spans from the neoplastic CIN3 core through to surrounding regions. Along this trajectory, the opposing expression gradients of key genes, such as *CDKN2A* and *IGF BP5*, reflect this correlation between oncogenic activity and immunosuppression. Secreted from stromal cells, *IGF BP5* has previously been known to trigger macrophage migration and may play a key role in macrophage processes and polarisation [72, 76]. In the context of CIN3 and cervical cancer, these findings suggest the role immune interactions may have on disease progression.

Our study identified coexpression of several ligand‐receptor pairs that may play crucial roles in cervical tumorigenesis and immune modulation. Ligand‐receptor analysis predicted a high number of cell–cell interactions in the immune cell cluster 10 but minimal interactions between this cluster and the neoplastic region. For instance, the *MDK*‐*SDC1* interaction, observed in our samples between the neoplastic cluster 8 and the adjacent cluster 9, has previously been reported as a prognostic biomarker [77]. *MDK*‐*TSPAN1* has been shown to promote tumorigenesis and chemoresistance in head and neck cancer [78, 79]. The statistically significant interactions using these ligand‐receptor pairs in CIN3 lesions suggests their potential involvement in cervical cancer development and immune evasion.

The distinct coexpression patterns of *IL34* and *CSF1R* suggest their involvement in tumour progression, immune suppression, and potentially macrophage polarisation. Although *IL34* was previously thought to be solely a secreted protein because it lacks transmembrane domains in its primary structure [13], Ogawa *et al* [80] discovered a membrane‐bound form of *IL34* through anti‐mouse *IL34* antibody staining. It was found that only cell‐surface *IL34*, and not the secreted variant, binds to the CSF1R receptor and induces the maturation of follicular dendritic cell‐induced monocytic cells [80]. This implies that the membrane‐bound form is the active form of *IL34*, and cell‐to‐cell contact is required to convey function. We observed a lack of coexpression of *IL34* and *CSF1R* within the neoplastic regions, despite high expression in the adjacent tissues. Additionally, there was an overall abundance of *CSF1R* compared to *IL34*, suggesting that macrophage infiltration in the neoplastic region occurs despite the absence of *IL34*. However, neoplastic regions of absent *IL34* were enriched primarily with M2 gene signatures, while adjacent regions rich in *IL34* expression were enriched with the M1 gene signature, suggesting that the lack of *IL34* may result in accumulation of immune‐suppressive macrophage phenotypes. This contrasts with the expression pattern of this ligand‐receptor pair observed in melanoma, where it has been described that heightened expression of *IL34* is related to lower immune cell frequencies, suggesting a more protumorigenic role [81, 82, 83]. This suggests a unique mechanism of action for HPV‐driven or squamous cell‐derived carcinogenesis. To assess the generalisability of this finding, we conducted additional analysis and observed parallels in *IL34*‐*CSF1R* expression between OPSCC and CIN3, suggesting similarities in HPV+ driven neoplasia regardless of anatomical site [24]. This highlights the potential for translational research across different types of squamous cell carcinomas.

Our study has several limitations, including a small sample size, and the lack of different CIN stages. These limitations may affect the generalisability of our findings and the interpretation of spatial and gene expression patterns. Current publicly available spatial datasets are highly limited. Although our findings displayed similar trends in an external dataset, future studies with larger cohorts, encompassing disease progression from normal cervical to cervical cancer and incorporating more diverse omics analyses are needed to validate and extend upon our observations. Despite the heterogeneity, our deep analysis of spatial transcriptomics data across the seven human CIN3 samples reveals consistent spatial patterns of the immune signatures within and adjacent to the precancer microenvironment. We found distinct and spatially defined interaction activities relative to locations with an active HPV+ signature. Together, the results suggest a potential strategy to modulate the immune system in HPV‐positive patients to prevent progression. This promising approach warrants further investigation.

## Author contributions statement

QN, IHF, JC and AC conceptualised the study. QN and AC supervised the study. GP, AC and HV performed data analysis, with interpretation and contributions from all authors. HV and ZX conducted the experiments. GP, HV and AC prepared visualisations. GP, HV, AC, QN and JC wrote the article, with contributions from all authors. BO and MW were responsible for case contribution and pathology analysis. All authors read and approved the final article.

## Supporting information


**Figure S1.** H&E images with pathological annotations outlining CIN3, SSC and non‐dysplastic squamous epithelium regions (non‐CIN3), p16 immunohistochemistry staining, coexpression of cervical cancer oncogenes (*CDKN2A*, *SERPINB3*, *TP63*, and *KRT5*) for all seven samples and spatial clustering
**Figure S2**. Quality control metrics across samples
**Figure S3**. Spatial gene expression of cervical cancer oncogenes (*CDKN2A*, *SERPINB3*, *KRT5*, and *TP63*) for all seven samples
**Figure S4**. Epithelial cell subclustering analysis reveals CIN3 specific regions
**Figure S5**. Spatial mapping of immune cell type signatures
**Figure S6**. Sub‐clustering of cluster 10
**Figure S7**. Pseudotime analysis identifies spatial gradient of CIN3 signature, mostly consistent with annotation, with some novel findings
**Figure S8**. Top 20 interacting ligand‐receptor pairs shown between clusters 8 and 9 and clusters 9 and 10 and all ligand‐receptor interactions between clusters 8 and 10
**Figure S9**. CIN3 associated suppression of IL34‐CSF1R co‐localisation identified in an external dataset
**Table S1**. List of marker genes used to identify IL34‐CSF1R interaction, canonical cervical oncogenes, CIN non‐CIN3, and SCC signatures
**Table S2**. List of marker genes used for immune cell type classification

## Data Availability

All analysis code is available at https://github.com/BiomedicalMachineLearning/CIN3. The spatial transcriptomics dataset generated in this study is deposited in UQ eSpace (https://espace.library.uq.edu.au/view/UQ:1bd2e07). Public datasets used include the OPSCC dataset (https://espace.library.uq.edu.au/view/UQ:698bb9e) and the HPV+ cervical dataset GSE208654 (https://www.ncbi.nlm.nih.gov/geo/query/acc.cgi?acc=GSE208654).
