# Supplementary material for: Spatial analysis of HPV‐associated cervical intraepithelial neoplastic tissues demonstrate distinct immune signatures associated with cervical cancer progression
Source: J Pathol. 2025 Dec 9;268(2):200–14. doi: 10.1002/path.70002 (PMC12805613; doi:10.1002/path.70002)
Supplement: Supplementary file 1 — Figure S1. H&E images with pathological annotations outlining CIN3, SSC and non‐dysplastic squamous epithelium regions (non‐CIN3), p16 immunohistochemistry staining, coexpression of cervical cancer oncogenes (CDKN2A, SERPINB3, TP63, and KRT5) for all seven samples and spatial clustering Figure S2. Quality control metrics across samples Figure S3. Spatial gene expression of cervical cancer oncogenes (CDKN2A, SERPINB3, KRT5, and TP63) for all seven samples Figure S4. Epithelial cell subclustering analysis reveals CIN3 specific regions Figure S5. Spatial mapping of immune cell type signatures Figure S6. Sub‐clustering of cluster 10 Figure S7. Pseudotime analysis identifies spatial gradient of CIN3 signature, mostly consistent with annotation, with some novel findings Figure S8. Top 20 interacting ligand‐receptor pairs shown between clusters 8 and 9 and clusters 9 and 10 and all ligand‐receptor interactions between clusters 8 and 10 Figure S9. CIN3 associated suppression of IL34‐CSF1R co‐localisation identified in an external dataset Table S1. List of marker genes used to identify IL34‐CSF1R interaction, canonical cervical oncogenes, CIN non‐CIN3, and SCC signatures Table S2. List of marker genes used for immune cell type classification [file PATH-268-200-s001.docx]

# **Spatial analysis of HPV-associated cervical intraepithelial neoplastic tissues demonstrate distinct immune signatures associated with cervical cancer progression**

# G Pavilion, H Vu *et al. J Pathol* <https://doi.org/10.1002/path.70002>

**Supplementary Figures S1–S9**

**Supplementary Tables S1–S2**

**
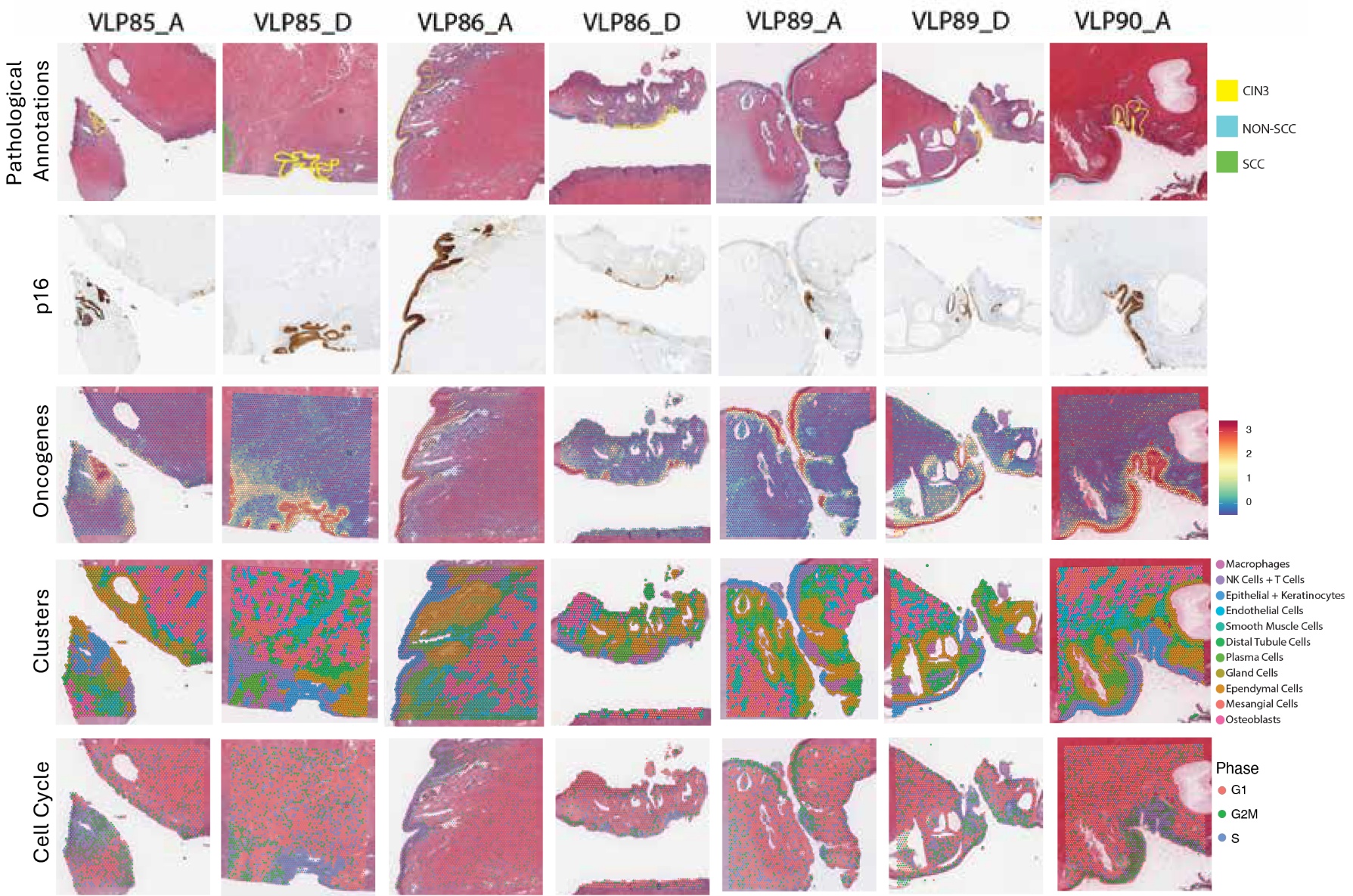
**

**Figure S1. H&E images with pathological annotations outlining CIN3, SSC and non-dysplastic squamous epithelium regions (non-CIN3), p16 immunohistochemistry staining, co-expression of cervical cancer oncogenes (*CDKN2A*, *SERPINB3*, *TP63*, and *KRT5*) for all seven samples and spatial clustering.**

**
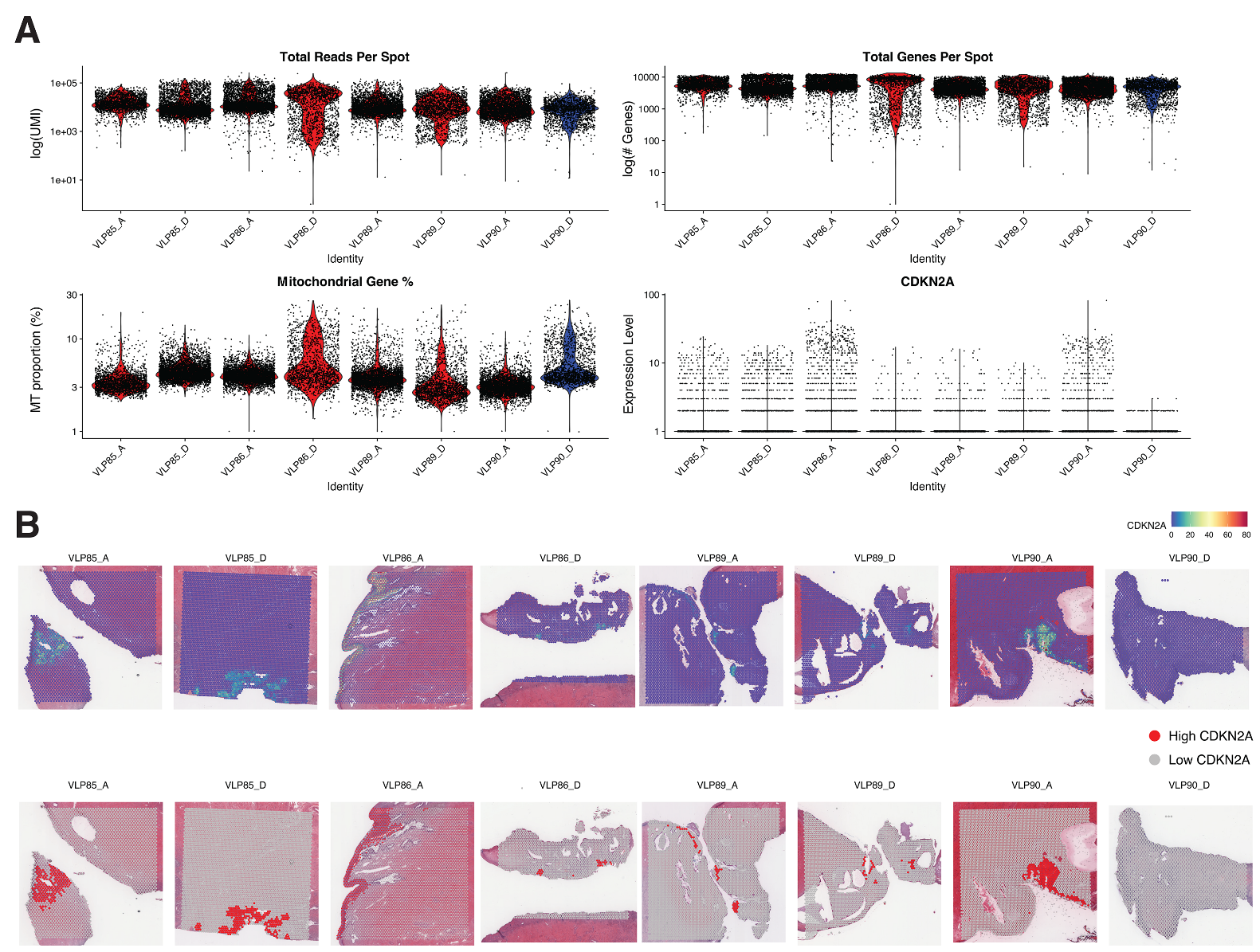
**

**Figure S2. Quality control metrics across samples**. (A) Violin plots showing total reads, total genes, percentage of mitochondrial and *CDKN2A* (p16) expression level per Visium spot for each sample. (B) Spatial visualization of *CDKN2A* (p16) expression across each sample with spots containing high and low *CDKN2A* expression highlighted in red and grey respectively in a corresponding plot below.


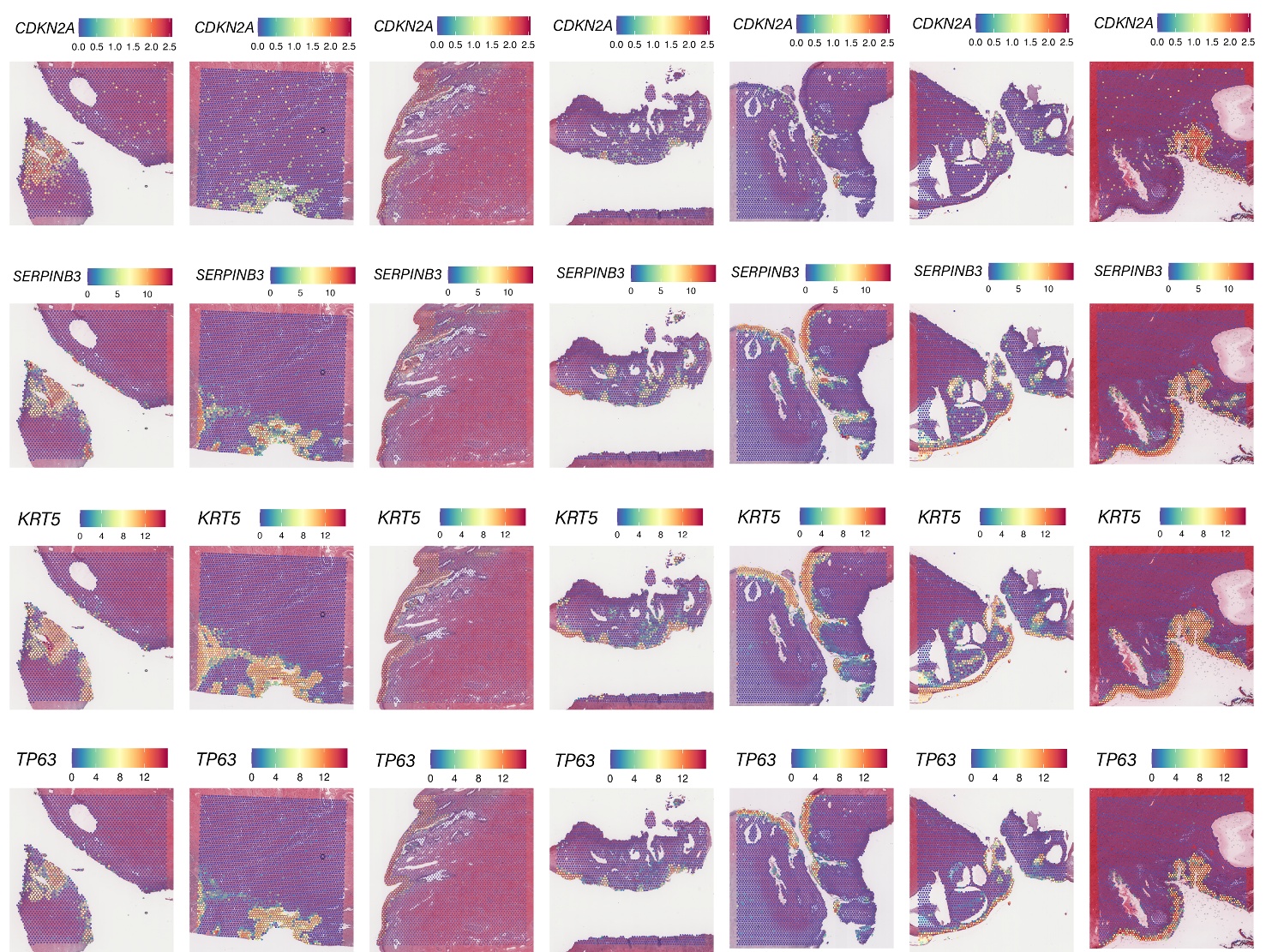


**Figure S3. Spatial gene expression of cervical cancer oncogenes (*CDKN2A*, *SERPINB3*, *KRT5*, and *TP63*) for all seven samples.**


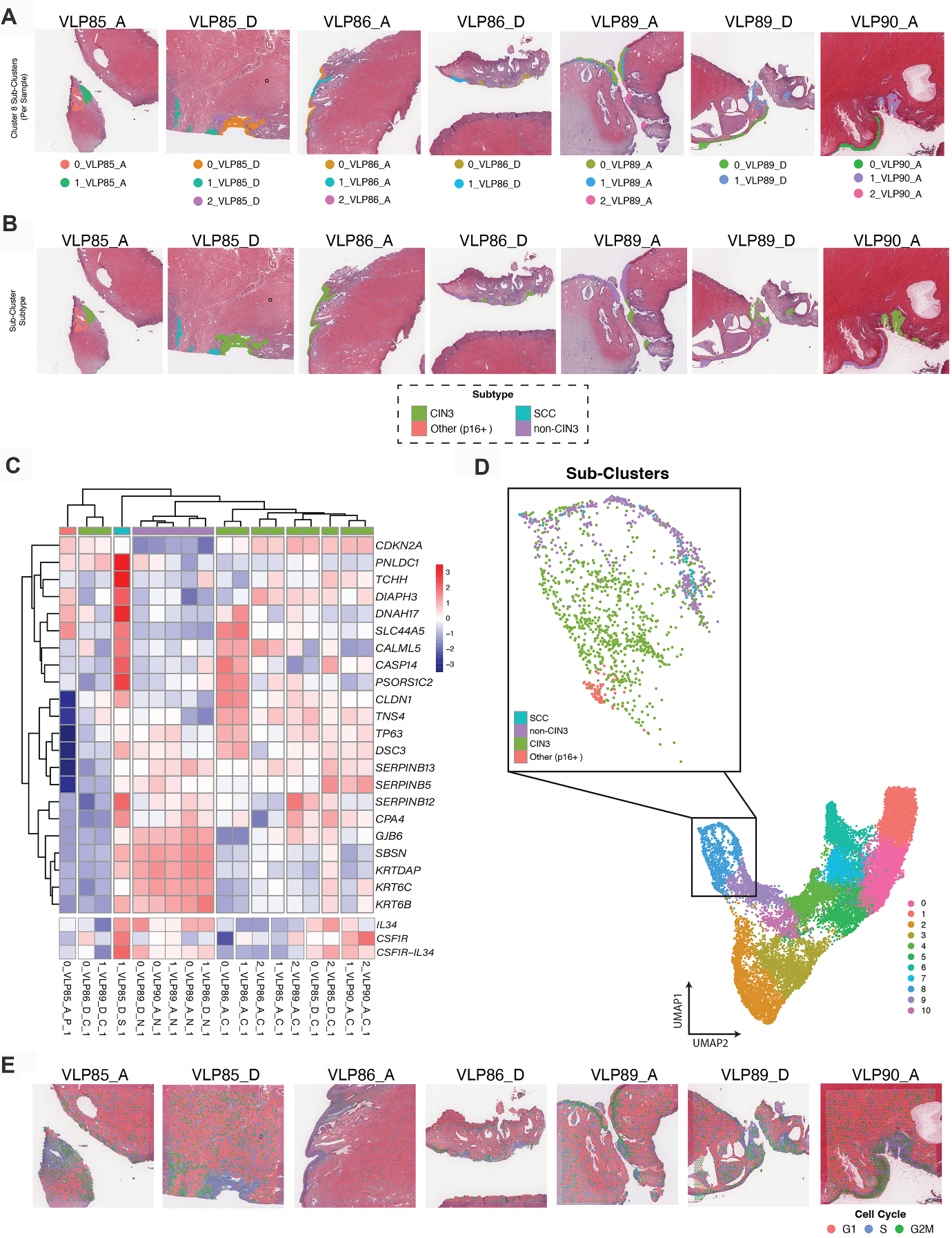


**Figure S4. Epithelial cell sub-clustering analysis reveals CIN3 specific regions.** (A) Sub-clustering results of the epithelial region identified at least two transcriptionally distinct populations for each individual sample. (B) Spatial representation of sub-cluster groupings into disease related subtypes based on transcriptional profiles (Cervical Intraepithelial neoplasia 3; CIN, non-squamous cell carcinoma; Non-SCC, non-squamous cell carcinoma p16+; Non-SCC (p16+), squamous cell carcinoma; SCC). (C) Pseudo-bulk expression heatmap of CIN3, non-SCC and SCC marker clustered hierarchically between individual sub-clusters. (D) UMAP embeddings displaying relative epithelial sub-types. (E) Cell cycle scoring analysis for all spots per sample.


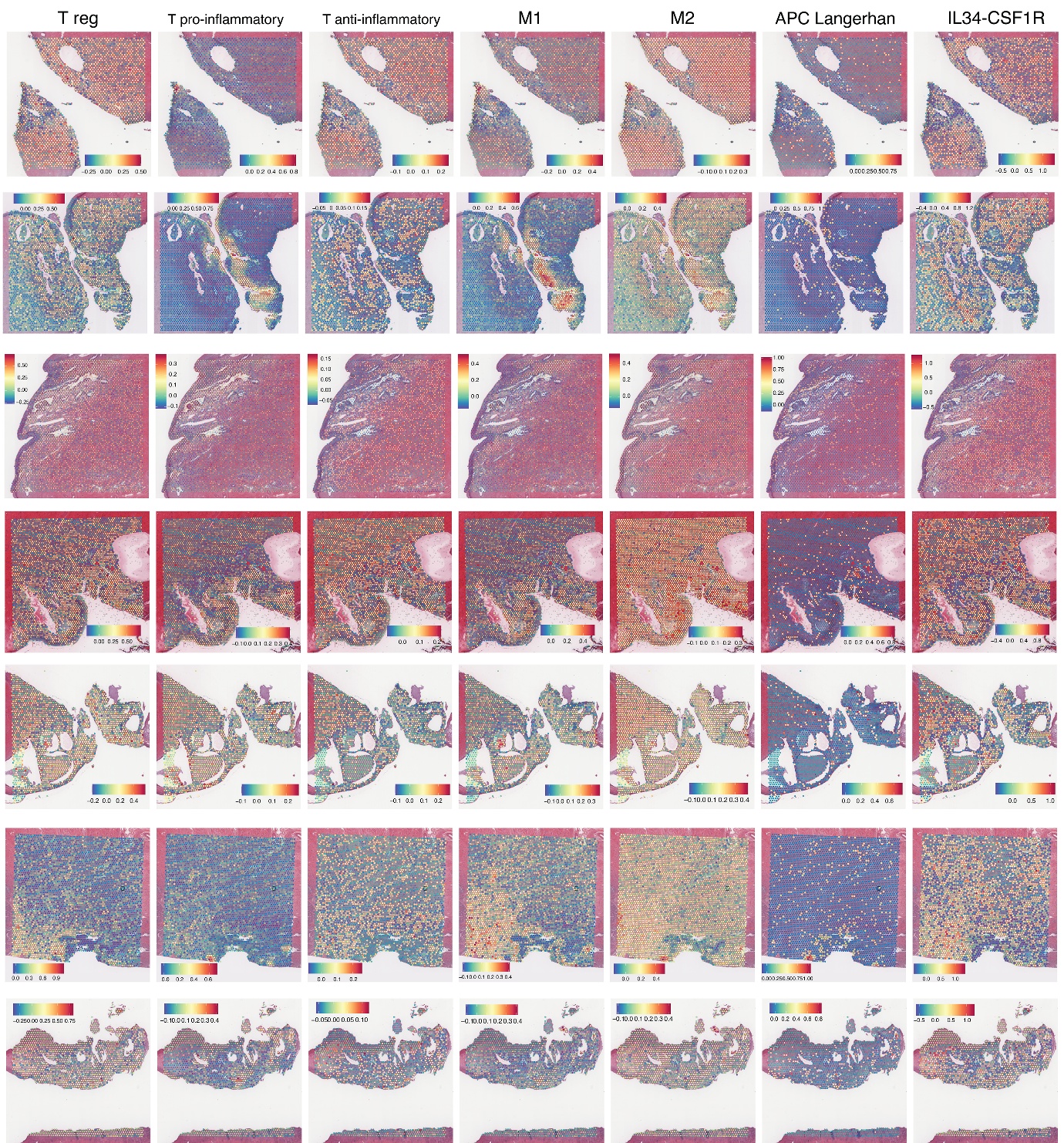


**Figure S5. Spatial mapping of immune cell type signatures.** From left to right, Geneset activity scores are shown for T regulatory, T pro-inflammatory, T anti-inflammatory, M1 macrophages, M2 macrophages, Langerhan Cells, and *IL34-CSF1R* co-expression for all seven samples. The list for these markers is shown in supplementary material, Table S1.


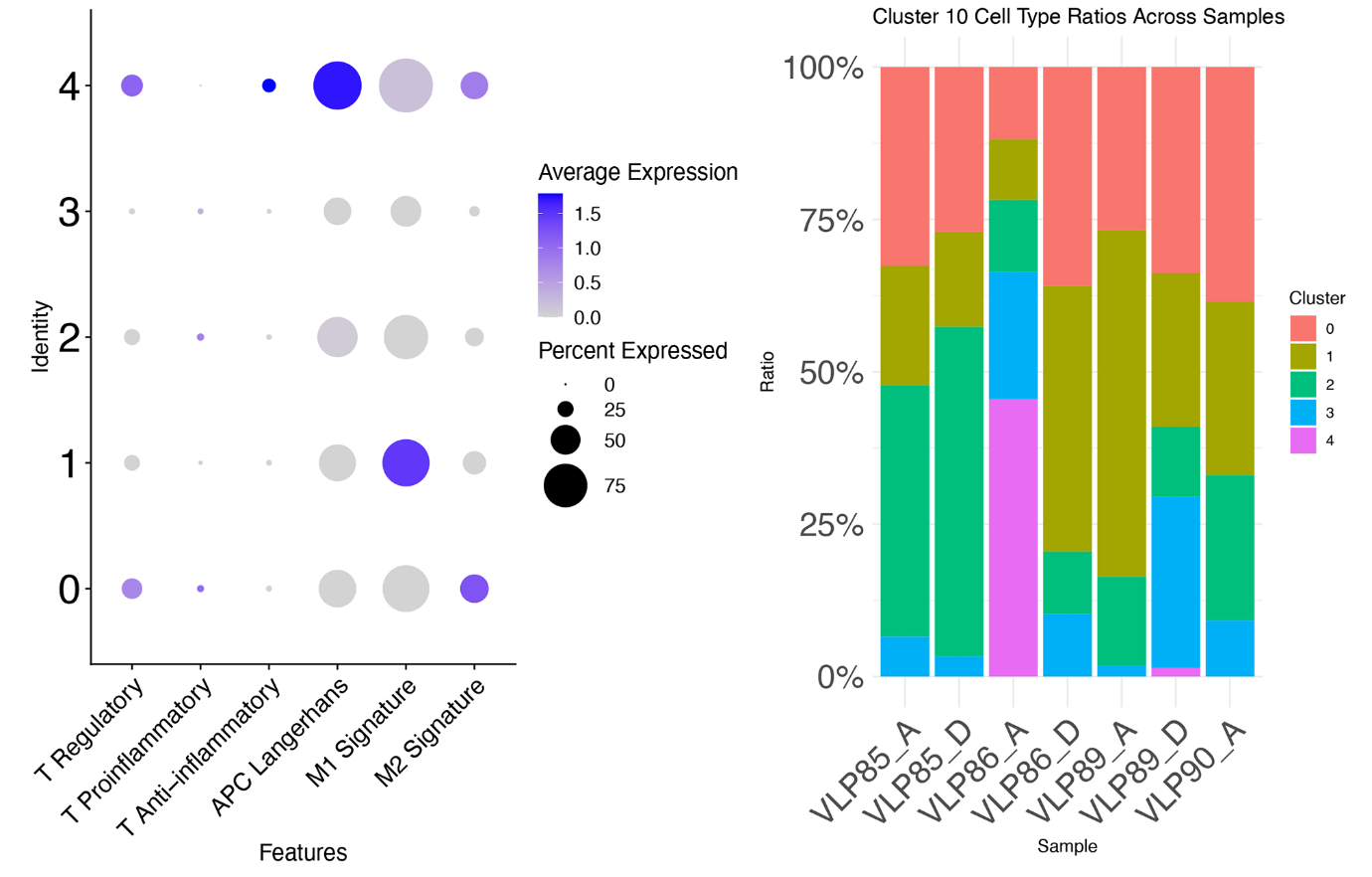


**Figure S6.** **Sub-clustering of cluster 10**. Immune cell type expression and cell type proportions for each sample. Clusters 0–4 representative of sub-clusters within cluster 10.


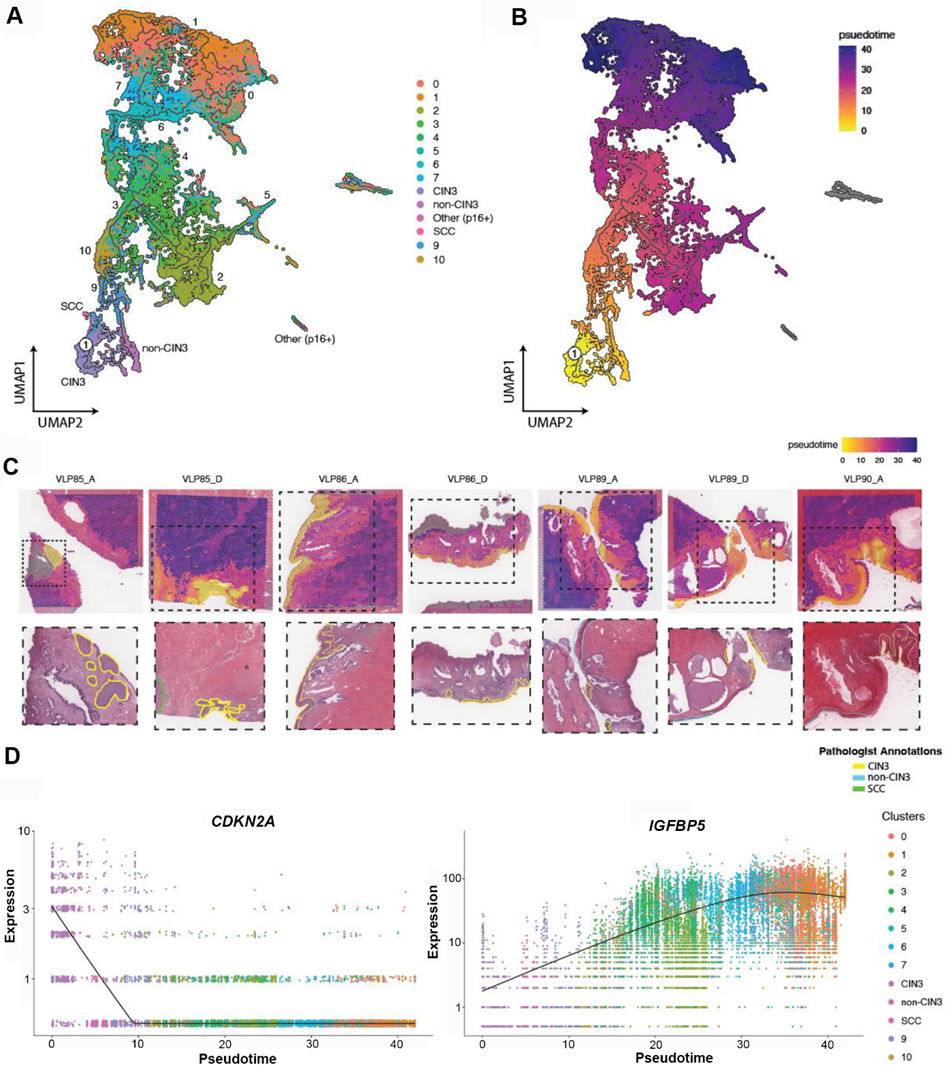


**Figure S7. Pseudotime analysis identifies spatial gradient of CIN3 signature, mostly consistent with annotation, with some novel findings.** (A) UMAP representation of trajectory path through all spots initialised from root 1 in a monocle trajectory landscape. (B) Pseudotime inferred from monocle 3 trajectory analysis, visualised with respect to epithelial cell subtypes defined by clusters shown in panel (A). (C) Spatial distribution of pseudotime trajectory plotted on to the tissue. (D) Expression patterns of *CDKN2A*, *CSF1R*, *IGFBP5*, and *IL34* across the spots ordered by the pseudotime trajectory.

**
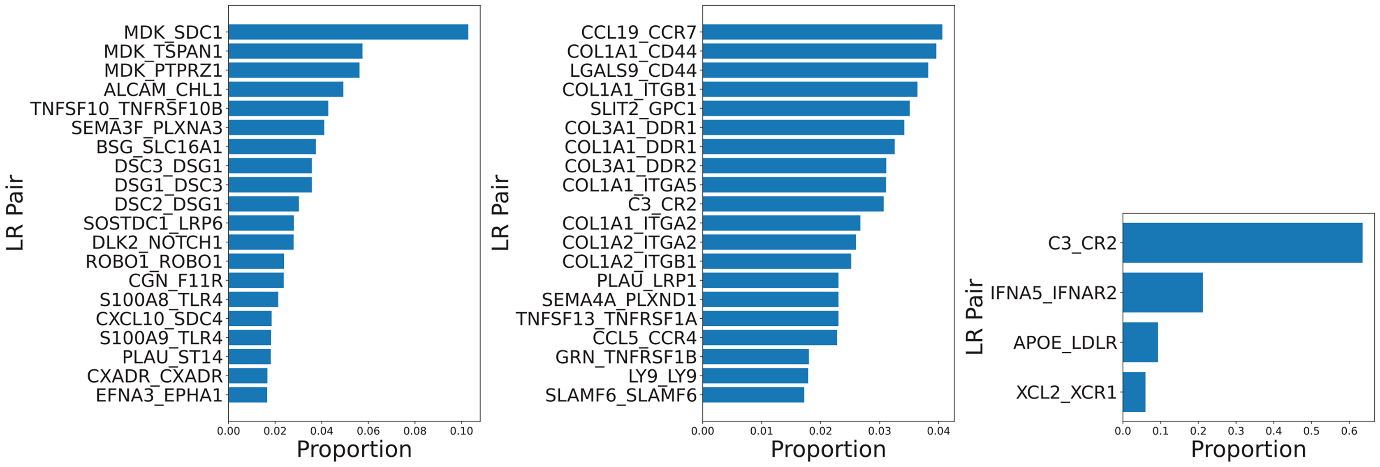
**

**Figure S8.** **Top 20 interacting ligand-receptor pairs shown between clusters 8 and 9 and clusters 9 and 10 and all ligand-receptor interactions between clusters 8 and 10.**


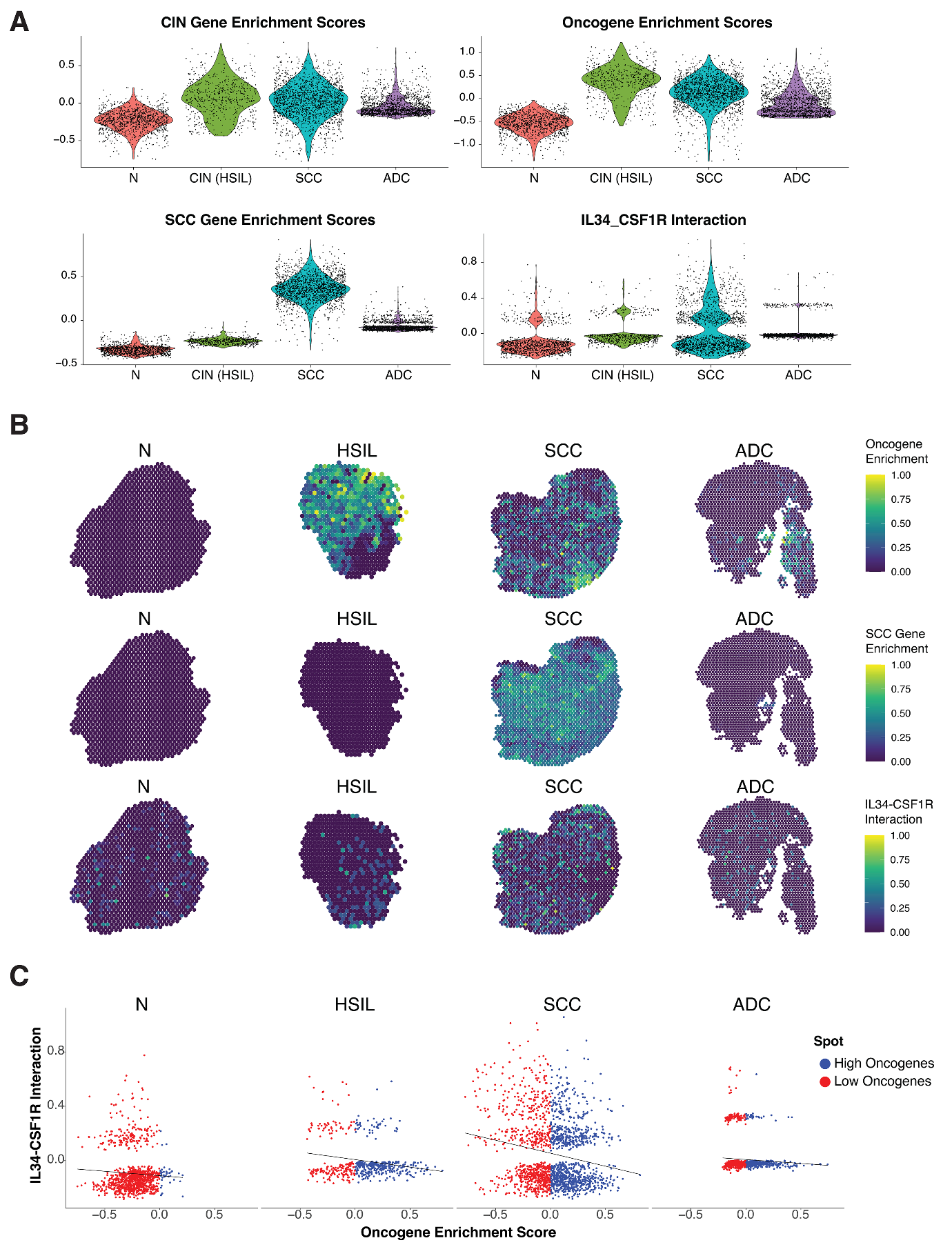


**Figure S9. CIN3 associated suppression of IL34-CSF1R co-localization identified in an external dataset.** (A) Violin plots showing the gene set enrichment scores of CIN, oncogenic and SCC markers compared to IL34-CSF1R coexpression analysis across normal (N), precancerous (HSIL) and cervical cancer (SCC and ADC) samples. (B) Spatial distribution of enrichment scores across each sample. (C) Correlation between IL34-CSF1R co-expression and oncogenic enrichment scores per spot across different diagnosis stages.

**Table S1**. List of marker genes used to identify *IL34*-*CSF1R* interaction, canonical cervical oncogenes, CIN non-CIN3 and SCC signatures.

| **Module Score Groups** | **Marker genes** |
| --- | --- |
| IL34-CSF1R | *IL34*, *CSF1R* |
| Cervical oncogenes | *SERPINB3*, *TP63*, *KRT5*, *CDKN2A* |
| CIN | *SERPINB13*, *SERPINB5*, *CLDN1*, *TP63*, *CDKN2A*, *TNS4*, *DSC3* |
| non-CIN3 | *KRT6C*, *GJB6*, *SBSN*, *KRTDAP*, *KRT6B* |
| SCC | *CASP14*, *PSORS1C2*, *DNAH17*, *SERPINB12*, *SLC44A5*, *TCHH*, *PNLDC1*, *DIAPH3*, *CPA4*, *CALML5* |

**Table S2**. List of marker genes used for immune cell type classification.

| **Immune cell type** | **Marker genes** |
| --- | --- |
| T-regulatory | *CD4*, *TNFRSF18*, *IL2RA*, *FOXP3*, *TGFB1*, *IL10* |
| T pro-inflammatory | *IL10*, *CSF3*, *IL1A*, *IL1B*, *IL6*, *IFNG*, *IL4*, *IL5*, *IL13*, *IL36B*, *IL36G*, *IL36A*, *CXCL8*, *TNF*, *IL18* |
| T anti-inflammatory | *IL10*, *IL12A*, *IL12B*, *IL22*, *IL37*, *IL1F10*, *TGFB1*, *IL4*, *IL11*, *IL13* |
| M1 macrophages | *CD80*, *IFNG*, *IL1B*, *IL6*, *TNF*, *CCL2*, *FCGR3A*, *FCGR2A*, *FCGR1A*, *IL12B*, *IL23A*, *MARCO*, *CD86* |
| M2 macrophages | *VTCN1*, *CD36*, *CD200R1*, *CD163*, *MRC1*, *CD209*, *CLEC10A*, *CLEC7A*, *CXCR1*, *CXCR2*, *MSR1*, *ARG1*, *NOS2*, *RETNLB*, *HMOX1*, *PPARG*, *CD274*, *IL1RN*, *TGM2*, *ACTG1*, *TIMP1*, *SPHK1*, *CXCL1*, *MERTK*, *ITGAV*, *IL1A*, *VEGFA*, *IVNS1ABP*, *CXCL8*, *DYSF*, *EHHADH*, *RIF1*, *LIG4* |
| Langerhans cells | *CD207*, *CD1A* |
